# Social, cultural and political conditions for advancing health equity: examples from eight country case studies (2011–2021)

**DOI:** 10.1136/bmjgh-2024-015694

**Published:** 2024-10-23

**Authors:** Miriam van den Berg, Joanne Flavel, Ashley Schram, Sharon Friel, Hailay Abrha Gesesew, Fran Baum

**Affiliations:** 1 Stretton Health Equity, School of Social Sciences, The University of Adelaide, Adelaide, South Australia, Australia; 2 Australian Research Centre for Health Equity, School of Regulation and Global Governance, Australian National University, Canberra, Australian Capital Territory, Australia; 3 Research Centre for Public Health, Equity and Human Flourishing (PHEHF), Torrens University Australia, Adelaide, South Australia, Australia

**Keywords:** Global Health

## Abstract

Progress in addressing systematic health inequities, both between and within countries, has been slow. However, there are examples of actions taken on social determinants of health and policy changes aimed at shaping the underlying sociopolitical context that drives these inequities.

Using case study methodology, this article identifies five countries (Ethiopia, Jordan, Spain, Sri Lanka and Vietnam) that made progress on health equity during 2011–2021 and three countries (Afghanistan, Nigeria and the USA) that had not made the same gains. The case studies revealed social, cultural and political conditions that appeared to be prerequisites for enhancing health equity.

Data related to population health outcomes, human development, poverty, universal healthcare, gender equity, sociocultural narratives, political stability and leadership, governance, peace, democracy, willingness to collaborate, social protection and the Sustainable Development Goals were interrogated revealing four key factors that help advance health equity. These were (1) action directed at structural determinants of health inequities, for example, sociopolitical conditions that determine the distribution of resources and opportunities based on gender, race, ethnicity and geographical location; (2) leadership and good governance, for example, the degree of freedom, and the absence of violence and terrorism; (3) a health equity lens for policy development, for example, facilitating the uptake of a health equity agenda through cross-sector policies and (4) taking action to level the social gradient in health through a combination of universal and targeted approaches.

Reducing health inequities is a complex and challenging task. The countries in this study do not reveal guaranteed recipes for progressing health equity; however, the efforts should be recognised, as well as lessons learnt from countries struggling to make progress.

**WHAT IS ALREADY KNOWN ON THIS TOPIC**

- Health inequities within and between countries are persistent. Despite the substantial body of evidence available to address these, progress has been slow and mixed.

**WHAT THIS STUDY ADDS**

- This study challenges the common perception that health inequities are too complex to address, by providing examples of health equity progress and the sociopolitical and cultural conditions that can facilitate this progress. In contrast, the study also identifies three countries that are struggling to advance health equity and highlights the conditions that are hindering progress.

**HOW THIS STUDY MIGHT AFFECT RESEARCH, PRACTICE OR POLICY**

- This study documents four key principles of practice that can advance health equity and illustrates, using practical examples, how certain social, cultural and political conditions can facilitate this agenda. Researchers, supported by a robust health equity monitoring system and in collaboration with policy-makers and public health practitioners, must continue to build evidence of how best to reduce health inequities. These collaborations must identify, evaluate and report on the effectiveness of actions aimed at reducing health inequities, so as to empower civil society and policy-makers to translate evidence into action.

## Introduction

 Despite global commitments to reducing health inequities,^1^ progress has been slow and mixed.^2^,^6^ The Commission on the Social Determinants of Health highlighted that ‘the social gradient in health within countries and the marked health inequities between countries are caused by the unequal distribution of power, income, goods and services, globally and nationally’.^7^ Staggering differences in life expectancy both between and within countries persist, and in some cases, are increasing.^8^,^10^ In contrast, progress in health equity (HE) is defined as ‘levelling the social gradient’ so that everyone can attain their full potential for health and well-being. HE progress is achieved through the systematic identification and elimination of inequities resulting from differences in social and commercial determinants of health.^11^

Evidence on how best to reduce health inequities is substantial and clear; however, the power and influence of political economies and their competing priorities have hampered progress.^5 12 13^ Researchers have explored the reasons behind inaction on HE, citing poor governance, globalisation, capitalism and corporate domination, competing vested interests, privatisation, repression of civil society, low unionisation, absence of collective bargaining, regressive welfare state policies, lack of understanding of how to act and low motivation.^512^,^14^ While a number of countries have given consideration to the social determinants of HE (SDHE), the recommendations of the Commission on Social Determinants of Health have not been significantly enacted.^10^

Reducing health inequities is often described as too challenging and complex.^15^ Yet while the need for studies that contribute to the evidence base for how to embed effective policies in specific political economy contexts is crucial,^16^ actions must not stall in the interim. Indeed, the European Health Equity Status Report^17^ stated that there is a need to change common perceptions that ‘health equity is too complex to address and that it is unclear what actions to take and which policies and approaches will be effective’.

With this in mind, this article identifies five countries that made progress on HE and three countries that had not made the same gains, during 2011–2021 (a period of 10 years after the launch of the Rio Political Declaration on Social Determinants of Health).^1^ We use the term ‘progress’ to mean a reduction in ‘within-country’ social gradients in health outcomes and ‘between country’ health inequities. On the basis of the cases, we identify a set of social and political conditions that appeared to be prerequisites for progress towards achieving HE.

## Method

This study applied comparative case study methodology—a qualitative method that allows researchers to explore events and activities across a defined period of time, taking into account the contextualised setting.^18 19^ We used an ‘explanatory’ approach as this allowed the research team to consider the ‘why’ and ‘how’ of progress on HE.^19^ The research question for the study was ‘What are the key country-level contextual conditions that may reduce health inequities, and the obstacles and challenges that prevent progress on HE?’

The number of case studies was determined in discussion with the funding body of the study (the WHO). Case selection commenced with a review of country-level data on inequities in child mortality, life expectancy and healthy life expectancy for the period 2011–2021. Using these data, accepted qualitative methods for selecting cases^20 21^ were used to develop a shortlist of potential countries that could offer insight into the mechanisms by which sociopolitical factors impact HE. The research team deliberated the shortlist, drawing on academic and grey literature, and using the theoretical lens of the Punching Above Weight Framework^22^ to ‘trace’ how within-country ‘processes’^21^ might either aid or harm HE. A final list of eight crucial country cases was determined—five countries that appeared to be making progress on HE (Ethiopia, Jordan, Spain, Sri Lanka and Vietnam) and three countries demonstrating evidence of lack of progress (Afghanistan, Nigeria and the USA).

Table 1 outlines selected descriptive variables for each country, and data on HE in under-5 mortality, life expectancy and healthy life expectancy, as examples of the data that were considered during the selection process. In addition to these HE indicator data, there were three main reasons why the eight countries were selected: (1) there was a substantial body of HE-related empirical evidence to draw on and major HE developments had been widely reported (eg, the significant reduction in poverty in Ethiopia, Vietnam’s universal health system, refugee programmes in Jordan); (2) several of the countries had been involved in armed conflict and other significant political events, likely to have major ramifications for HE and (3) the countries were balanced across country income groups, as well as WHO regions.

**Table 1:** Countries included in the study

Country		Population (2020)	WHO region	Income group	Child mortality (deaths/1000 live births, 2021) compared with income country group	Life expectancy at birth for both sexes (years, 2021) compared with income country group	Healthy life expectancy at birth for both sexes (years, 2019) compared with income country group
Poor progress	Afghanistan	39 million	Eastern Mediterranean	Low	46 LIC: 47	62 LIC: 62	53.9 LIC: 56.7
	Nigeria	206 million	African	Lower middle	71 LMIC: 35	53 LMIC: 67	54.4 LMIC: 60
	United States of America	329 million	The Americas	High	5 HIC: 4	76 HIC: 79	66.1 HIC: 69.8
Making progress	Ethiopia	115 million	African	Low	35 LIC: 47	65 LIC: 62	59.9 LIC: 56.7
	Jordan	10 million	Eastern Mediterranean	Upper middle	13 UMIC: 11	74 UMIC: 75	67.6 UMIC: 67
	Sri Lanka	22 million	South-East Asia	Lower middle	6 LMIC: 35	76 LMIC: 67	67 LMIC: 60
	Spain	47 million	European	High	3 HIC: 4	83 HIC: 79	72.1 HIC: 69.8
	Vietnam	98 million	Western Pacific	Lower middle	17 LMIC: 35	74 LMIC: 67	65.3 LMIC: 60

Child mortality (under 5 years) and life expectancy data were sourced from World Bank.^112^ Healthy life expectancy data were sourced from WHO Health Equity Monitor.^38^ Data for each country are compared with income groups.

HIC, high-income countries; LIC, low-income countries; LMIC, lower-middle-income countries; UMIC, upper-middle-income countries.

For each selected country, we undertook a rapid review^23^ of quantitative and qualitative data sourced from academic and grey literature. Examining credible government and non-government reports, academic literature, and media reports is considered suitable for ascertaining historical and current states of HE.^24^ Search terms were related to the key variables outlined in the Punching Above Weight Framework and data/publication dates were 2011–2021. The Google search engine was used to identify the country social and economic indicators and ranking indices and long-term trends including the Gini coefficient, Universal Health Care (UHC) Service Coverage Index,^25^ Gender Inequality Index (GII),^26^ Sustainable Development Goals (SDGs),^27^ Democracy Index (DI),^28^ Multidimensional Poverty Index,^29^ Human Development Index (HDI),^30^ World Governance Indicators (WGIs),^31^ Global Peace Index (GPI),^32^ Global Gender Gap Index (GGI)^33^ and Social Protection Rating (SPR).^34^ We took guidance from Barros *et al*^35^ and Penman-Aguilar *et al*^24^ on measuring HE advancement and recommended data sources, including further interrogation of the SDGs progress reporting. Further searches using academic databases (Web of Science, ProQuest and Medline) and Google were conducted to identify grey literature, to gain an understanding of country contextual factors.^36^ For each country case, key independent variables related to the context (political and institution; economic, social, environmental and health policies; cultural and societal norms and values), civil society and democracy, and SDH (based on the Punching Above Weight Framework^22^ were summarised into good (✓), moderate (◯) or poor (✖) matrices.^37^ Further detail about the approach is provided in online supplemental file A. Eight country case studies summarising the available quantitative and qualitative data were prepared. Each country case study was analysed deductively using the key domains of Baum *et al*’s HE framework and revealed four key conditions for reducing health inequities.

### Patient and public involvement

Patient and public involvement was not appropriate for this study as no new patient data were collected.

### Findings

For each country case study, we documented evidence of the social gradient in selected health outcomes. We commence this section by outlining an example of these data. Next, we present our ratings for five indicators of action on the SDHE; followed by ratings for eight sociopolitical and cultural indicators. We combine our ratings of quantitative variables with qualitative data from the literature.

### Social gradient in health

**Figure 1:**
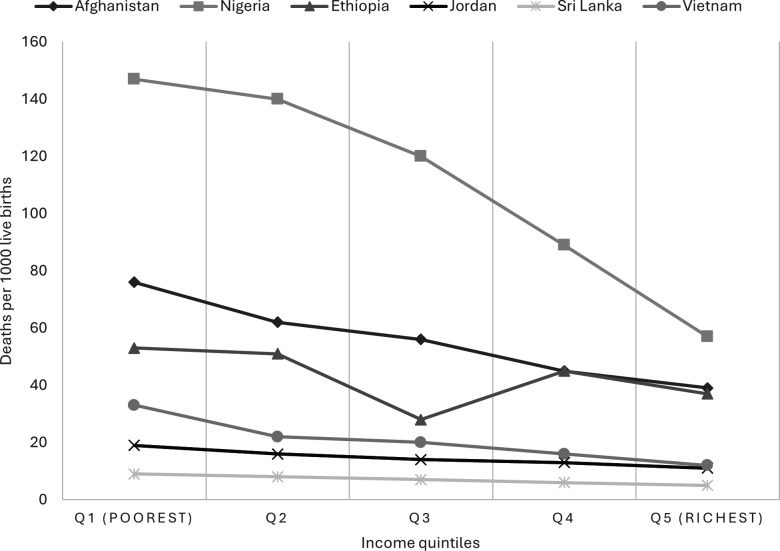
Estimated child mortality (deaths per 1000 live births, 2021), by income quintiles, selected countries.

For each country case study, evidence of the social gradient in health was recorded. An example of these data is shown in figure 1, which demonstrates the steepness of the social gradient in child mortality for six countries (data were not available for the USA or Spain).^38^ Inequities in child mortality were most pronounced in Nigeria (which recorded a gap of 90.8 deaths between the richest and poorest quintiles: Q1: 56.6–Q5: 147.4) and Afghanistan (the gap was 37.2, Q1:75.7–Q5:38.5), while steepness of the gradient was lowest in Jordan (gap was 7.8, Q.1: 10.9–Q.5: 18.7) and Sri Lanka (the gap was 4.4, Q.1: 4.6–Q.5: 9.0). Child mortality was much higher than the average for Sub-Saharan Africa in Nigeria, and despite an overall decline between 2008 and 2018, the gap in child mortality between the poorest quintile and the wealthiest quintile increased from 2.5 times the mortality rate of the wealthiest quintile in 2008 to 3.2 times the mortality rate of the wealthiest quintile in 2018.^39^ Among countries making HE progress, such as Ethiopia, the gap in child mortality between the poorest quintile and the wealthiest quintile decreased between 2016 and 2021 from 23 deaths to 16 deaths per 1000 live births.^40 41^ In Sri Lanka, there were no significant differences in child mortality by residence or wealth quintiles in 2016.^42^

In the case of missing data for Spain and the USA, HE data were available through other sources. For example, in the USA, child mortality among non-Hispanic black people is double the rate for non-Hispanic white people.^43^ Inequities in life expectancy among racial/ethnic groups were widespread and enduring, with Native Americans having the lowest life expectancy across all races. This group also experiences higher rates of numerous health problems, for example, the rate of serious psychological distress among adult Native Americans is 2–5 times higher than the prevalence for other racial/ethnic groups.^43^ Health inequities between population groups within countries were evident on the basis of wealth, income, gender, race, rurality and education.^3843^,^46^

### Indicators of SDHE

Table 2 outlines our ratings for five indicators of action on SDHE-related quantitative variables: human development, poverty, UHC, gender equality and progress on the SDGs.

**Table 2:** Progress rating of five selected health equity indicators (action on social determinants of health equity for eight countries (2011–2021)

Country		Punching above weight variables: social determinants of health equity				
		Human Development Index	Poverty (Multidimensional Poverty Index)	Universal Healthcare	Gender Inequality Index	Progress on Sustainable Development Goals
Poor progress	Afghanistan	✖	**Ο**	**Ο**	✖	✖
	Nigeria	✖	**Ο**	✖	✖	✖
	USA	**✓**	**✓**	**✓**	**Ο**	**Ο**
Making progress	Ethiopia	✖	✖	✖	**Ο**	✖
	Jordan	**✓**	**✓**	**✓**	✖	**Ο**
	Spain	**✓**	**✓**	**✓**	**✓**	**Ο**
	Sri Lanka	**✓**	**✓**	**✓**	**Ο**	✖
	Vietnam	**✓**	**✓**	**✓**	**Ο**	**Ο**

Relative ratings: **✓**=good; Ο=moderate; ✖=poor (refer to online supplemental file A).

HDI=Human Development Index (2019).^30^ MPI=Multidimensional Poverty Index (various-2020).^29^ UHC=Universal Health Coverage (2021).^25^ GII=Gender Inequality Index (2021).^26^ SDGs=Sustainable Development Goals.^52^

Despite having a far higher Gross National Income (GNI),^47^ human development in Nigeria was much lower than in Sri Lanka and Jordan, which had substantially lower GNI.^30^ In 2019, Nigeria’s Inequality-adjusted HDI (IHDI) ranking (measured on a scale of 0–1 and reflecting the distribution of three dimensions of human development: a long healthy life, being knowledgeable and having a decent standard of living) had increased less than 0.06 since 2010 (from 0.283 in 2010 to 0.341 in 2021). Nigeria’s IHDI reflected significant sex, education, economic status and urban–rural inequities.^30 48^ Among the countries making HE progress, poverty rates had declined during 2011–2021 (eg, Vietnam, Ethiopia),^49^,^51^ though in a number of cases entrenched gaps between population groups remained.^50^,^54^

The relative ratings outlined in table 2 provided a snapshot of the state of selected HE-related conditions; however, additional qualitative data provided further insight into key contextual developments. For example, the USA recorded a high score for UHC.^55^ However, it is noted that the USA is the only high-income country in the world without a universal publicly funded health system.^56^ Americans spend over twice as much on healthcare per person compared with other high-income countries on average, with healthcare expenditures growing from 5% of GDP in 1960 to almost 18% in 2018.^57^ In 2020, 28 million Americans did not have health insurance, and the proportion of uninsured children and young people living in poverty had increased from 1.6% in 2018 to 9.3% in 2020.^58^

Gender equity was found to be a greater priority in countries that were advancing an HE agenda, although, across the board, progress is slow. Spain, which was ranked 14th globally in the GGI,^33^ had achieved gender parity in educational attainment, and health and survival, and had progressive maternity and social security policies for women in the workplace.^59^ In Jordan, gender parity had been achieved in literacy and education participation for children aged 6–15 years,^33^ and the government had established high-level processes to elevate women’s status in the country.^60^ Jordan, however, ranked poorly on the global stage for gender equality (GII), though it was fifth among countries that make up the Middle East and North Africa region.^33^ Afghanistan was ranked 156 out of 156 countries with its lowest score being for political empowerment.^33^

All countries reported varying levels of progress on the SDGs. Two out of three countries making poor HE progress were also making poor progress in relation to the SDGs. Of the better-performing countries, Spain, Jordan and Vietnam were making the most progress on the SDGs. Examples of country cases that were more obviously progressing the SDGs agenda included participation in voluntary national SDGs reviews (eg, Jordan) and actively seeking collaborative solutions to global challenges (eg, Vietnam).^61^,^64^

### Social and political conditions

The sociopolitical context of each country provided insight into the structural conditions impacting HE progress. In table 3, we rate HE progress in relation to selected sociopolitical variables for each country. We drew on academic and grey literature to gain insight into these indices—summarising the main findings below. Pivotal to interpreting the relative ratings in this table are the timeline under consideration (2011–2021) and the dynamic nature of a country’s sociopolitical conditions. We note in particular the changing state of political stability, violence and ethnic discrimination in Ethiopia, Sri Lanka and Afghanistan. In addition, our timeline would barely have documented the impact of the COVID-19 pandemic.

**Table 3:** Evidence of selected sociopolitical conditions as prerequisites for health equity progress for selected countries (2011–2021)

Country		Punching above weight variables: politics, policies, culture and civil society engagement for Health equity							
		Historical sociocultural conditions	Political stability and leadership	Good governance (World Governance Indicator)	Peace and low involvement in armed conflict Global Peace Index and Sustainable Development Goals (SDG16))	Democracy Index	Willingness to engage and collaborate	Universal and proportionately targeted policies for daily living (Social Protection Rating)	Action on climate change (SDG13)
Poor progress	Afghanistan	✖	✖	✖	✖	✖	✖	✖	**✓**
	Nigeria	✖	✖	✖	✖	**Ο**	✖	**Ο**	**Ο**
	USA	✖	**Ο**	**Ο**	✖	**Ο**	**✓**	**Ο**	✖
Making progress	Ethiopia	**Ο**	**Ο**	✖	**Ο**	✖	✖	**Ο**	**✓**
	Jordan	Ο	**✓**	**✓**	**Ο**	✖	✖	**Ο**	**Ο**
	Spain	**✓**	**Ο**	**✓**	**✓**	**✓**	**✓**	**✓**	✖
	Sri Lanka	**Ο**	**Ο**	**✓**	**Ο**	✖	**Ο**	**Ο**	**✓**
	Vietnam	**Ο**	**✓**	**✓**	**✓**	✖	✖	**Ο**	**Ο**

Relative ratings: **✓**=good; Ο=moderate; ✖=poor (refer to online supplemental file A).

WGI=World Governance Indicators.^31^ GPI=Global Peace Index.^32^ SDG16=Sustainable Development Goal 16–Peace, Justice and Strong Institutions.^27^ DI=Democracy Index.^28^ SPR=Social Protection Rating (2015–2016).^34^ SDG13=Sustainable Development Goal 13–Climate action.^52^

### Historical sociocultural conditions

There was evidence that historical sociocultural narratives, norms and values are deeply embedded in societies, manifesting as ‘rules’ that generate social hierarchies with implications for HE. For example, in the USA, inequities in SDH such as education, income/wealth and housing are heavily contextualised by racism. Opportunities and outcomes for black Americans have been profoundly shaped by the legacy of slavery (1526–1865) and racial segregation (1877–1963). Within the USA, ‘fourth-grade failure syndrome’ refers to a bias in schools (eg, cultural insensitivity, disproportionately harsh discipline and lowered teacher expectations) that has the cumulative effect of diminishing black students’ (especially boys’) enthusiasm and motivation for school.^65^

Similar pathologies were evident in relation to gender-based inequities. In Vietnam, despite many gender equity gains (eg, in education), deeply embedded cultural norms contributed to progress in other areas (eg, property ownership, participation in political activity) being slow or even regressive. Traditional rigid gender perceptions of values and roles remained firmly sustained across all Vietnam’s social strata, stifling women’s advancement and well-being.^66^

In Spain, attempts had been made to rewrite harmful historical, cultural narratives relating to slavery, black communities and human rights violations.^67^,^69^ In 2014, Spain enacted laws prohibiting racial profiling and racial discrimination, and local civil society groups have been actively involved in monitoring and advocating against police racial profiling.^67^

### Political stability, leadership and governance

Political volatility, inconsistent leadership and governance problems appeared to be detrimental to advancing an HE agenda in many of the case studies (eg, USA, Afghanistan, Nigeria, Sri Lanka and Ethiopia). Low levels of trust in institutions and political parties, deep dysfunction in government, increasing threats to freedom of expression and a degree of societal polarisation that impedes consensus were evident in the USA during the 2011–2021 period.^28 70^

All three countries struggling to make HE progress were ranked lower than their income group counterparts on the WGIs.^31^ Nigeria performed poorly in comparison to other low-middle-income countries on all six aggregate governance indicators (voice and accountability, political stability and absence of violence/terrorism, control of corruption, rule of law, government effectiveness, regulatory quality). The USA ranked lower on political stability and absence of violence/terrorism stability, and voice and accountability.

Afghanistan has a long history of political instability and ineffective governance, as well as armed conflict, and despite population health improvements when foreign forces and international aid agencies had a stronger presence in the country, the return of a Taliban government in 2021 instilled uncertainty about the likelihood of continued HE advancement.^71^ In contrast, relatively stable political leadership in a tumultuous region saw the delivery of a long-term vision for socioeconomic development that included an analysis of health indicators arising from social and economic progress in Jordan^72 73^ and in Ethiopia^74^ prior to the civil war.

### Peace and absence of armed conflict

The highly destructive HE consequences of armed conflict were evident in several countries. In Ethiopia, where despite HE progress over the previous decade, civil war erupted in the northern state of Tigray in 2020, devastating gains were made in maternal and child health, family planning and chronic disease management.^75^ Afghanistan and Nigeria were also deeply involved in armed conflict during the 2011–2021 period. The Boko Haram insurgency in Nigeria resulted in Northeast Nigerians experiencing protracted displacement, loss of income and declining agricultural production.^76 77^ Sri Lanka experienced 30 years of civil war until 2009 and has since continued to struggle with reconciliation postconflict.^78^

According to the GPI (2021), Spain and Vietnam had a ‘high’ state of peace, and Jordan had a ‘medium’ state, and all three countries had improved their ranking in previous years. The USA, Sri Lanka and Ethiopia had all deteriorated in their standing. There was no change in Afghanistan, with the state of peace ranked the lowest out of 163 countries.^32^ Similarly, all three countries making poor progress (USA, Nigeria and Afghanistan) recorded ‘stagnating’ progress in relation to SDG 16: Peace, Justice and Strong Institutions.^52^

### Democracy

The DI takes into account the electoral process and pluralism, government functioning, political participation, political culture and civil liberties.^28^ While Spain had a ‘full democracy’, Sri Lanka and the USA’s democracy was described as ‘flawed’, Nigeria had a ‘hybrid regime’ and Afghanistan, Vietnam, Ethiopia and Jordan had ‘authoritarian regimes’. Jordan’s reduced social gradient in child mortality (illustrated in figure 1) appears to have occurred within the context of the country’s socially oriented monarchy.^79^

### Willingness to engage and collaborate

The CIVICUS monitor collates data on the state of civil society and civic freedoms in 196 countries.^80^ None of the countries in this study were fully ‘open’. Afghanistan, Nigeria, Ethiopia and Jordan were ‘repressed’, that is, civic space was significantly constrained; and the USA and Sri Lanka were ‘obstructed’, that is, civic space was heavily contested by power holders. Vietnam was ‘closed’. Afghanistan and the USA have experienced dramatic declines in freedom over the past 10 years.^81^ Spain was ranked higher by CIVICUS and described as ‘narrowed’ because while the state allowed individuals and civil society organisations to exercise their rights to freedom of association, peaceful assembly and expression, violations of these rights also took place.^80^

The study found that there are often discrepancies between policy rhetoric and meaningful reality. For example, the authoritarian Jordanian Government has given consideration to greater citizen participation in decision-making and increased transparency and accountability across all levels of government,^82^ with the King outlining his recognition of the ‘vital role that civil society plays in enhancing our democratic model’.^83^ Despite promising in-principle progress, researchers continue to express concern about the underpinning neopatriarchal structure and functioning of Jordanian society, which seems to cycle between liberalisation and control.^84 85^

### Universal and proportionately targeted policies for daily living

The SPR assesses the quality of a country’s current policy and institutional framework, where ‘quality’ refers to how conducive that framework is to fostering poverty reduction, sustainable growth and the effective use of development assistance.^34^ Across the eight country cases, the SPR was highest for Ethiopia, Jordan and Spain, and lowest for Afghanistan and the US. Universal health and social protection policies provide a solid platform for HE progress. Prior to the war in Tigray, Ethiopia had some of the fastest rates of poverty reduction in the world and ran the largest social protection programme in Africa.^74^ While in the USA, the social protection system has been described as involving eligibility rules that ‘are too strict’, with benefits that ‘are too hard to access even when people are eligible’ and benefits amounts that ‘are too low’. The social protection system in the USA is reported to contribute to widening inequities along the lines of race and gender.^86^

### Action on climate change

Countries making progress on HE had recognised climate change as a major challenge and progressed policy responses. All the countries making HE progress had signed the Paris Climate Agreement and participated in the UN Climate Change Conference in Glasgow (COP26). In 2017, Vietnam, for example, aimed to reduce greenhouse gas emissions by 20% from the agriculture and rural development sector by 2020.^63^ Ethiopia demonstrated regional leadership in setting a climate change action agenda^87^ and Sri Lanka developed a National Climate Change Policy in 2012.^88^ Sri Lanka and Ethiopia were reported to be on track to achieving SDG 13: Climate Change.^52^ Although Spain has recently taken some steps towards addressing climate change, for example, the adoption of the Climate Change and Energy Transition Law,^89^ as with many high-income countries, progress has been slow and major challenges remain.^52 90^

## Discussion

Reducing health inequities between and within countries has been a long-term goal of the WHO and many of its member states^91^ and will be the focus of the upcoming WHO World Report on SDHE. In 2008, when the WHO Commission on Social Determinants of Health handed down its final report, it concluded that ‘Social injustice is killing people on a grand scale’.^7^ These social injustices include the social norms, policies and practices that tolerate or promote unfair distribution of and access to power, wealth and other necessary resources, which in turn affect opportunities for health. That researchers are reporting on widening health inequities is a major concern.^92^ However, as this study has shown, there are also examples of progress in some countries.

Assessing countries on the basis of their progress in reducing health inequities is challenging for many reasons.^24 36^ Health inequities are systematic, arising from the complex interplay of variables that encapsulate politics, policies, civil society and culture. The interplay of factors underlying HE cannot be measured directly as the concept is closely tied to fairness and social justice, and social norms, which vary across societies.^24^ The sociopolitical context within which health inequities arise is dynamic, as seen in the cases of Afghanistan and Ethiopia, with both countries returning to a state of political instability and increasing violence towards the end of 2020/2021. This makes it challenging to capture the HE progress snapshot.

With these factors in mind, it is important to highlight the limitations of the study reported in this article. We undertook a rapid review of academic and grey literature on eight countries using an HE lens. It was beyond the scope of the study to attempt to account for all of the innovations that were occurring across society to address structural and intermediary factors that are the origins of inequities in health. Even though our review yielded a large volume of data, as mentioned above, HE progress and the measurement thereof is a moving feast. Societies are continually evolving, drawing on the past and forging new futures, and as such, it is challenging to showcase all the factors that might impact a country’s performance at a point in time, particularly given time lags in data collection, analysis and the publication of research findings. We recognise the limitations of individual socioeconomic indices,^93^ hence we included a wide range of indicators. It is not routine practice for non-health data to be collected for the purpose of monitoring HE. As such, data sources for some variables are not consistent across all countries. Across the board, within-country HE data is insufficient, hence many of the variables considered socioeconomic and political conditions, rather than quantitative population health data, and we strengthen our argument by applying the Punching Above Weight Framework.^22^ We note that there may be multiple valid approaches to operationalising the concept of HE within different social contexts. The countries selected all provided examples of actions that could progress or undermine HE progress; however, there are other country-level examples that could also be explored.

While recognising these limitations, what these country case studies have illustrated are four key contextual factors that can help advance HE. Table 4 summarises these prerequisites for progressing HE and lists key policy action area that will support these goals.

**Table 4:** Prerequisites for progressing HE and key policy recommendations

**Action directed at structural determinants** Reduce multidimensional poverty. Build HE-focused social protection systems that respond to life-course stages. Ensure poverty reduction strategies are implemented proportionately to reduce HE gaps. Build HE-focused universal healthcare systems. Develop and implement long term mechanisms to advance human development in relation to daily living needs (eg, housing, income), education and healthcare. Advance gender equity across all areas of society. Take a zero tolerance approach to discrimination on the basis of class, gender, race and other identity markers.	**Apply an HE lens to policy formulation and implementation** Institutionalise HE assessment processes through legislation to implement in public policy sectors. Promote examples of effective outcomes arising from the use of HE assessment processes. Find a common language and understanding of HE so that it becomes more palatable for those in non-health sectors. Build cross-sector alliances and foster leadership to advance action on the SDH outside of the health sector. Use the Sustainable Development Goals as a mechanism to achieve HE.
**Advocate for good governance** Ensure that HE is integrated into responses to war and conflict. Build strong governance frameworks in relation to voice and accountability, political stability and absence of violence/terrorism, government effectiveness, regulatory quality, rule of law and control of corruption. Close the gender equity gap in leadership roles. Establish mechanisms for greater accountability for leaders to advance HE.	**Reduce social gradients in health and increase health equity** Implement HE policies and programmes to a scale and intensity that matches levels of need. Monitor and assess progress against social gradients in health. Gather and publish HE data for all countries. Gather and report HE data at all levels of government within countries and facilitate HE evidence-based decision-making.

HE, health equity; SDH, social determinants of health.

First, action directed at structural determinants is central to HE progress. Many of the conditions that facilitated the advancement of an HE agenda across the five country cases making HE progress were structural in nature, that is, they related to historical, political and institutional conditions, governance, policies, culture, geography and democracy.^22^ Structural oppression is an act of ‘violence’^92^ that leads to the inequitable distribution of SDH across population groups.^94^ Such discrimination operates across multiple social domains, including income, housing, education, gender, race and employment, to create and perpetuate the oppression of some groups over others and was evidenced in the USA’s social and health protection systems,^86^ Afghanistan’s treatment of women and girls,^95^ and Sri Lanka’s discrimination against Tamil peoples.^96^ Acts of violence rest within social institutions and are reinforced through political systems of governance^94^ and may present as being difficult to change; but data that illustrate shallower social gradients in health outcomes (eg, in Jordan, Sri Lanka and Vietnam) demonstrate that change is possible.

Based on the case studies presented here, action directed towards poverty reduction, increasing the HDI, providing UHC, and adherence to the principles of good governance must be prioritised in order to achieve HE. To reduce health inequities, poverty reduction policies must be implemented proportionately to ensure racial, age, social class (education and occupation) and gender gaps do not persist.^97 98^ Inequities in health have a major impact on a country’s HDI rating. For example, in the African region, nearly 90% of African countries lose more than 25% of their HDI when inequalities are taken into account.^99^ A large body of evidence demonstrates that HE gains arise from governmental investment in the conditions of daily life, education and health. Access to UHC, even in low HDI countries, can help reduce health inequities.^7^

Less attention has been given to the concept of good governance for HE—our second condition for HE advancement. Political leaders are important for advancing HE because they can influence agendas, advance collaborations that drive intersectoral policy, foster political will and become stewards for HE.^100^,^102^ So many of the conditions for HE could be accelerated into action by the tenets of good governance: voice and accountability, political stability and absence of violence/terrorism, government effectiveness, regulatory quality, rule of law and control of corruption. Percival *et al*^103^ note that ‘Leaders have missed opportunities to build more equitable and resilient economic, social and political systems’. Political leaders and governments have the power to shape the social fabric needed to overcome the challenges and barriers to HE, including armed conflict and violence. Addressing HE can independently influence levels of peace.^103^ Further, increasing the participation of women in government leadership roles improves the quality of governance.^104^

Much has been written about the value of using a health equity lens for policy development, particularly for advancing HE through non-health policy agendas.^105 106^ Countries making progress on HE had included not only SDH but sociopolitical variables (such as those related to the voice of civil society) into strategic policies. For this reason, we recognised countries employing cross-sectoral approaches to health as the third prerequisite for advancing HE. For example, the long-term vision of Ethiopia’s 2010 5-year Growth and Transformation Plan was ‘to become a country where democratic rule, good governance and social justice reigns, on the involvement and free will of its peoples’ and to ‘extricate itself from poverty’ and ‘become a middle-income country’.^107^ The creation of fairer society, with access to adequate income, employment and housing, was an important component of Sri Lanka’s Vision 2025.^108^ Good health, along with its many determinants—at the level of active and empowered citizens, and efficient and effective government—were explicitly named as among the desired outcomes of Jordan’s 2025 strategic plan.^73^ While such directives may not be framed as HE policies, they are pivotal for advancing this agenda because they demonstrate high-level leadership and influence access to SDH through cross-sector agendas. When governments institutionalise HE in this way, population health outcomes improve and the needs of those in the most vulnerable circumstances are addressed.^103 109^ Advancing the SDGs provides an important opportunity to advance a health-equity-in-all-policies approach.^110^

The final prerequisite for advancing HE identified through the country case analysis was the need for actions that will reduce the steepness of the social gradient in health. Researchers have called for a combination of universal and targeted approaches for levelling social gradients in health outcomes.^111^ A number of countries in this study had demonstrated significant reductions in poverty across the population; however, closer investigation revealed persistent pockets of disadvantage. An important component of this prerequisite for HE progress is an appropriate, robust and long-term monitoring system for HE.^7 94^ The WHO HE Monitor is a step in that direction.

## Conclusion

Countries making progress on HE demonstrated examples of how, through a combination of sociopolitical actions, it is possible to bring HE research evidence, social structures and policies, and civil society more into alignment. By no means are their journeys universal or guaranteed recipes for progressing HE in all contexts; however, the efforts should be recognised, along with lessons learnt from failures.

## Supplementary material

10.1136/bmjgh-2024-015694 online supplemental file 1

## Data Availability

Data are available in a public, open-access repository. Data may be obtained from a third party and are not publicly available. All data relevant to the study are included in the article or uploaded as online supplemental information.
