# Supplementary material for: Social, cultural and political conditions for advancing health equity: examples from eight country case studies (2011–2021)
Source: BMJ Glob Health. 2024 Oct 23;9(Suppl 1):e015694. doi: 10.1136/bmjgh-2024-015694 (PMC11664353; doi:10.1136/bmjgh-2024-015694)
Supplement: online supplemental file 1 [file bmjgh-9-Suppl_1-s001.pdf]

Supplementary A

Table 2 Notes:

- The Human Development Index (HDI) reflects achievement against three key dimensions: a long healthy life, being knowledgeable and having a decent standard of living (UNDP, 2020). Country data were rated as < 0.550 = poor (✖), 0.551-0.699 = moderate (O), ≥ 0.700 = good (✓). These ratings were adapted from the HDI ranking approach: < 0.550 = low, 0.550-0.699 = medium, 0.700 ≥ 0.799 = high, and ≥ 0.800 = very high (UNDP, 2020).
- The Multidimensional Poverty Measure (MPM) considers access to monetary resources, education and basic infrastructure and ranges from 0 to 1, with higher values implying higher multidimensional poverty (OPHI & UNDP, 2021). Countries within the range 0-0.333 were rates as good (✓), countries within the range 0.334-0.667 were rated moderate (O) and countries within the range 0.668-1.0 were rate as poor (✖). These rates are based on the 1/3 weighting scale used to calculate the MPM (Alkire & Santos, 2011).
- Universal Health Coverage (UHC) is a unitless scale (0-100) that considers the provision of essential health services, from health promotion to prevention, treatment, rehabilitation and palliative care (WHO & World Bank, 2021). We used the WHO rating system, which considers countries scoring >60 to have good coverage (✓), countries with a score of 40-59 to have moderate coverage (O) and countries with a score less than 39 to have poor coverage (✖).
- The Gender Inequality Index (GII) is a composite measure, reflecting inequality in achievements between women and men in three dimensions: reproductive health, empowerment and the labour market (UNDP, 2022). In line with country grouping in the GII data files, we rated countries according to their ranking within their HDI grouping (very high, high, medium and low development). Countries within the range 0-33.3% were rated as good (✓), countries within the range 33.34-66.67% were rated moderate (O), and countries within the range ≥66.68 were rated as poor (✖).
- The Sustainable Development Goals includes 17 goals agreed by all UN member states (UN, 2015). We assessed country progress as reported on the UN Country Profiles website (UN, 2021). Countries were rated as good (✓) on the basis of trend data

demonstrating the country was on track or maintaining SDG achievement in respect of most the 17 goals; moderate (○) if a country was moderately improving with respect to most of the goals; or poor (✖) if progress had stagnated or was decreasing.

Table 3 notes:

- To assess historical sociocultural conditions, we drew on qualitative data from academic and grey literature. We rated countries as good (✓) for multiple powerful examples of actions aimed at changing harmful historical sociocultural narratives and institutions, moderate (○) if a small number examples were identified or poor (✖) if no examples were identified.
- In assessing political stability and leadership we drew on the WGI (as described below): Political Stability and Absence of Violence/Terrorism. We also drew on academic and grey literature on political movements and looked for evidence of leadership for health equity (e.g. use of health equity-related terminology) on official government websites and in policy documents. We rated countries as good (✓), moderate (○) or poor (✖) on the basis of comparing the data to the average for countries in the same income group across the world (i.e. good=above the group average, moderate=similar to the group average; poor=below the group average).
- World Governance Indicators (WGI) include six aggregate governance indicators: Voice and accountability, political stability and absence of violence/terrorism, government effectiveness, regulatory quality, rule of law and control of corruption. was used to rate countries for governance performance (Kaufmann & Kraay, 2021). The WGI scores range from -2.5 (poor governance) to +2.5 (better governance). We rated countries as good (✓), moderate (○) or poor (✖) on the basis of comparing the data to the average for countries in the same income group across the world (i.e. good=above the group average, moderate=similar to the group average; poor=below the group average).
- In terms of rating peace and low involvement in armed conflict, we obtained data from the Global Peace Index (Institute for Economics & Peace, 2021) and reflected on SDG16, as well as academic and grey literature on countries' involvement in wars and the associated consequences for health. The Global Peace Index ranks countries in terms of very high-high-medium-low-very low state of peace, which we adapted to ✓=very high or high, ○=medium, ✖ = low or very low. SDG16 includes 16 indicators. In our rating, we considered how many of these indicators presented ongoing challenges to a country, as well as the overall rating awarded to the country for SDG16.

- The Democracy Index (DI) provides a snapshot of the state of democracy around the world, and is based on five categories: electoral process and pluralism, the functioning of government, political participation, political culture, and civil liberties (The Economist Intellegence Unit, 2020). Countries classified as full democracy were rated (✓), flawed democracies or hybrid regimes: (O) and authoritarian regimes: (✖).
- The DI as described above provided guidance for rating a country's willingness to engage and collaborate. We further searched academic and grey literature for examples of citizen engagement in the policy process, and cross-country collaborations. We rated countries as good (✓) for a DI civil liberties score of 7.56-10 and evidence of powerful examples of engagement and collaboration, moderate (O) for a DI civil liberties score of 4.56-7.55, accompanied by a small number of examples of engagement and collaboration, or poor (✖) for a DI civil liberties score of 0-4.55, with few or no examples identified.
- The CPIA Social Protection Rating (1: low-6: high) captures government policies in social protection and labour market regulations that reduce the risk of becoming poor, assist those who are poor to better manage further risks, and ensure a minimal level of welfare to all people (World Bank, 2021). We considered country scores on the basis of comparing the data to the average for countries in the same income group across the world. No data were available for the US, Spain and Jordan. We therefore drew on academic and grey literature for examples of universal and proportionately targeted policies for daily living.
- We based our rating for action on climate change on SDG13 data (UN, 2021). Countries that had achieved the SDG or were on track were rated as good (✓), countries where challenges or significant challenges remain were rated moderate (O), and countries where major challenges remain were rated poor (✖).
